# A call to establish HLA registry in Iraq

**DOI:** 10.1016/j.jtumed.2023.07.009

**Published:** 2023-07-31

**Authors:** Mahmood D. Al-Mendalawi

**Affiliations:** Pediatrics and Child Health, Consultant Paediatrician, Department of Paediatrics, Al-Kindy College of Medicine, University of Baghdad, Baghdad, Iraq

## Dear Editor,

When the first human leucocyte blood group antigen was discovered by Dausset in 1958, nobody could anticipate that was the beginning of a new and exceedingly fruitful research field in human medicine. Firstly, it became clear that this and a large number of other leucocyte antigens belong to the same genetic system. This system is the human leukocyte antigen (HLA) system, which acts as a primary barrier in organ transplantation. It was later discovered, which was even more surprising, that HLA is heavily involved in the development of a group of diseases.^1^

The HLA system is determined by genes located on the short arm of chromosome no. 6. The HLA loci constitute a genetic region called the major histocompatibility complex (MHC). The MHC encompasses genes (including HLA) essential to the integrity of the immune response. The HLA antigens have the function of controlling self-recognition and defending against pathogens.^2^ By virtue of their wide polymorphisms, the HLA loci guarantee that few individuals are identical and the population at large is well-armed to respond to the attack by pathogens. Each individual has a distinct HLA set. As some HLA antigens are present on different body tissues rather than only blood cells, their detection is defined as “Tissue Typing” or “HLA typing”.^3^

The HLA system's clinical relevance extends beyond infectious diseases to include autoimmune disorders and organ transplantation.^3^ The precise mechanisms underlying the majority of HLA-disease associations are poorly understood. The development of social stratification centered on religion, language, and geography, impacts of founder effect, ethnic mixture or selection pressure brought on by environmental factors are some of the mechanisms suggested for such variation.^4^

The HLA frequencies vary widely across populations. Demographic factors and selection are suggested to have figured HLA variations across continents.^5^ The polymorphisms of various HLA genes and haplotypes in different racial and geographical populations are vital in the evaluation of a particular population's genetic characteristics, detection of HLA-matched unrelated hematopoietic stem cell transplantation donors, and conducting disease association studies.^6^ Accordingly, HLA typing has become an effective tool to define the suitability of matching donors and recipients for organ transplantation, predicting the risk of developing certain diseases, including autoimmune diseases.^3^

The first study on HLA in Iraq dates back to 1977 when population HLA frequency was reported.^7^ A cascade of studies on HLA has followed, however, most of them involved the association between HLA and certain diseases. In 2013, wider research on the association of HLA alleles and different diseases in different ethnic groups in Iraq was released. It revealed that the HLA typing of Iraqi and non-Iraqi populations differed in some ways. It was thought that these diseases could be brought on by a variety of racial, religious, and environmental factors.^8^ Various efforts were devoted thereafter to launch a nationwide study on setting allelic and haplotypic HLA map and their correlation with diseases but regrettably, they were not successful. Iraq is among Arabian countries where consanguineous marriage is culturally the preferred type of marriage. This form of marriage is thought to increase the risk of different hereditary diseases and some of which are closely linked to certain HLA alleles. The last published annual statistical report released in Iraq by the Ministry of Health and Environment in 2022^9^ pointed to the increased prevalence of various HLA-associated diseases such as celiac disease, diabetes mellitus, and autoimmune thyroid diseases. Moreover, the growing health issues of chronic kidney diseases and leukemia are expected to increase the need for kidney and bone marrow transplantation. We believe that the establishment of the HLA registry in Iraq is deemed critical to have a database on the allelic and haplotypic HLA distribution, HLA-associated disorders, and hematopoietic stem cell or organ donor selection. It could also help plan certain therapeutic and preventive measures. The sustaining framework for updating and a critical review and validation of tools used in registry-related activities are of the utmost importance. We believe that there are certain barriers that mitigate that project, namely limited awareness of the importance of that project, particularly among policymakers, physicians, and researchers as well as limited financial resources. A call is sent to policymakers and HLA typing researchers to consider establishing an HLA registry in Iraq to build up a national database that could be used to predict disease susceptibility and resistance, assist in matching organ donors, and control genetic diversity in a manner similar to that employed in developed countries.^10^, ^11^, ^12^

## Source of funding

The author did not receive any specific grant from funding agencies in the public, commercial, or not-for-profit sectors.

## Conflict of interest

The author has no conflict of interest to declare.

## Ethical approval

Not applicable.

## Author's contribution

The author designed the manuscript, reviewed the literature, wrote and approved the final draft, and is responsible for the content and similarity index of the manuscript.
